# Adenylosuccinate lyase in pancreatic ductal adenocarcinoma chemoresistance: from purine metabolism to metabolic vulnerability

**DOI:** 10.20517/cdr.2026.58

**Published:** 2026-08-24

**Authors:** Tung-Wei Hsu, Chih-Ming Su, Yen-Hao Su, Po-Hsiang Liao, Charupong Saengboonmee, Wan-Yu Wang, Hsin-An Chen

**Affiliations:** ^1^Department of Surgery, Division of General Surgery, Shuang Ho Hospital, Taipei Medical University, New Taipei City 23561, Taiwan.; ^2^Department of Surgery, Division of General Surgery, School of Medicine, College of Medicine, Taipei Medical University, Taipei 11031, Taiwan.; ^3^TMU Research Center of Cancer Translational Medicine, Taipei Medical University, Taipei 11031, Taiwan.; ^4^TMU Research Center for Digestive Medicine, Taipei Medical University, Taipei 11031, Taiwan.; ^5^Department of Surgery, School of Medicine, College of Medicine, Taipei Medical University, Taipei 11031, Taiwan.; ^6^Metabolic and Weight Management Center, Shuang Ho Hospital, Taipei Medical University, New Taipei City 23561, Taiwan.; ^7^Center for Drug Research and Development, College of Human Ecology, Chang Gung University of Science and Technology, Taoyuan 333324, Taiwan.; ^8^Department of Biochemistry, Faculty of Medicine, Khon Kaen University, Khon Kaen 40002, Thailand.; ^9^Cho-Kalaphruek Excellent Research Project for Medical Students, Faculty of Medicine, Khon Kaen University, Khon Kaen 40002, Thailand.; ^10^Cholangiocarcinoma Research Institute, Khon Kaen University, Khon Kaen 40002, Thailand.

**Keywords:** Adenylosuccinate lyase, pancreatic ductal adenocarcinoma, purine metabolism, metabolic reprogramming, chemoresistance, therapeutic targeting

## Abstract

Pancreatic ductal adenocarcinoma (PDAC) is among the most lethal human malignancies and is characterized by profound metabolic reprogramming and marked therapeutic resistance. Among the metabolic enzymes that sustain tumor fitness, adenylosuccinate lyase (ADSL) has emerged as a critical regulator of *de novo* purine biosynthesis with broader roles in oncogenic signaling, metabolic adaptation, and chemoresistance. ADSL catalyzes two key reactions in purine synthesis, contributing to adenylosuccinate-to-adenosine monophosphate (AMP) production, fumarate generation, and regulation of intermediates such as succinylaminoimidazole carboxamide ribotide (SAICAR), which functionally intersects with glycolysis and transcriptional control. In PDAC, dysregulation of ADSL-associated metabolism has been linked to aggressive tumor biology and altered therapeutic responses. Mechanistically, ADSL may modulate gemcitabine responsiveness through context-dependent effects on purine nucleotide homeostasis, replication-stress adaptation, redox regulation, and ferroptosis-associated signaling. Beyond its canonical biosynthetic function, ADSL-related metabolic flux may support adaptive survival through SAICAR–pyruvate kinase M2 (PKM2) signaling, glycolytic remodeling, and fumarate-associated pathways that influence redox homeostasis, epigenetic regulation, and stress-response signaling. These interconnected mechanisms position ADSL as a context-dependent metabolic vulnerability in chemoresistant PDAC. Here, we summarize current understanding of ADSL biology and discuss its prognostic, mechanistic, and therapeutic relevance in PDAC chemoresistance, with emphasis on gemcitabine resistance, replication stress buffering, and metabolic adaptation. Targeting ADSL-associated metabolic dependencies may provide a promising strategy to disrupt the metabolic circuitry underlying PDAC aggressiveness and drug resistance.

## INTRODUCTION

Pancreatic ductal adenocarcinoma (PDAC) remains a formidable clinical challenge because of its aggressive behavior, early metastatic dissemination, and profound resistance to current therapies^[1,2]^. Despite substantial advances in cancer biology and drug development, PDAC continues to rank among the deadliest malignancies, with a 5-year relative survival rate of approximately 13.7% based on recent SEER data from 2016-2022^[3]^. The PDAC microenvironment, characterized by hypoxia, nutrient deprivation, and dense desmoplastic stroma, imposes severe metabolic constraints on tumor cells^[4,5]^. To survive and proliferate under these conditions, PDAC cells undergo extensive metabolic rewiring, a hallmark process that fuels tumor progression and therapeutic resistance.

Among the metabolic adaptations that support PDAC growth, nucleotide biosynthesis is particularly important because it sustains proliferation, preserves DNA replication fidelity, and supports RNA transcriptional output^[6,7]^. Within this framework, adenylosuccinate lyase (ADSL) occupies a central position in *de novo* purine synthesis by catalyzing two critical reactions: the conversion of adenylosuccinate to adenosine monophosphate (AMP) and fumarate, and the cleavage of succinylaminoimidazole carboxamide ribotide (SAICAR) to aminoimidazole carboxamide ribotide (AICAR) and fumarate^[8,9]^. These reactions replenish intracellular purine pools while simultaneously linking nucleotide synthesis to broader metabolic circuits, including the tricarboxylic acid (TCA) cycle through fumarate production^[10]^. Although ADSL deficiency has classically been studied in the context of inherited metabolic disease, its dysregulation in cancer suggests functions that extend beyond housekeeping metabolism and may intersect with oncogenic signaling and adaptive stress responses.

Beyond ADSL, multiple enzymes involved in *de novo* purine biosynthesis may contribute to the metabolic fitness and treatment tolerance of PDAC cells^[6,11,12]^. Upstream enzymes such as phosphoribosyl pyrophosphate amidotransferase (PPAT), glycinamide ribonucleotide transformylase (GART), phosphoribosylformylglycinamidine synthase (PFAS), phosphoribosylaminoimidazole carboxylase/phosphoribosylaminoimidazolesuccinocarboxamide synthase (PAICS), and 5-aminoimidazole-4-carboxamide ribonucleotide transformylase/inosine monophosphate (IMP) cyclohydrolase (ATIC) collectively support IMP generation and thereby sustain nucleotide availability for DNA replication, RNA synthesis, and repair of therapy-induced DNA damage^[7,11]^. Downstream enzymes involved in AMP and guanosine monophosphate (GMP) branch pathways may further shape the balance between adenine and guanine nucleotide pools, which can influence replication stress responses and cellular adaptation to antimetabolite-based chemotherapy^[9,12]^. However, compared with other purine biosynthetic enzymes, ADSL is positioned at a particularly important metabolic branch point because it catalyzes both the SAICAR-to-AICAR and adenylosuccinate-to-AMP reactions, thereby linking purine nucleotide production with SAICAR-associated pyruvate kinase M2 (PKM2) signaling and fumarate-associated redox, epigenetic, and immunometabolic regulation^[8,13]^. Therefore, although purine biosynthesis should be viewed as an integrated metabolic network, ADSL represents a mechanistically distinctive metabolic node that may influence therapeutic responsiveness and chemoresistance in PDAC.

**Figure 1:**
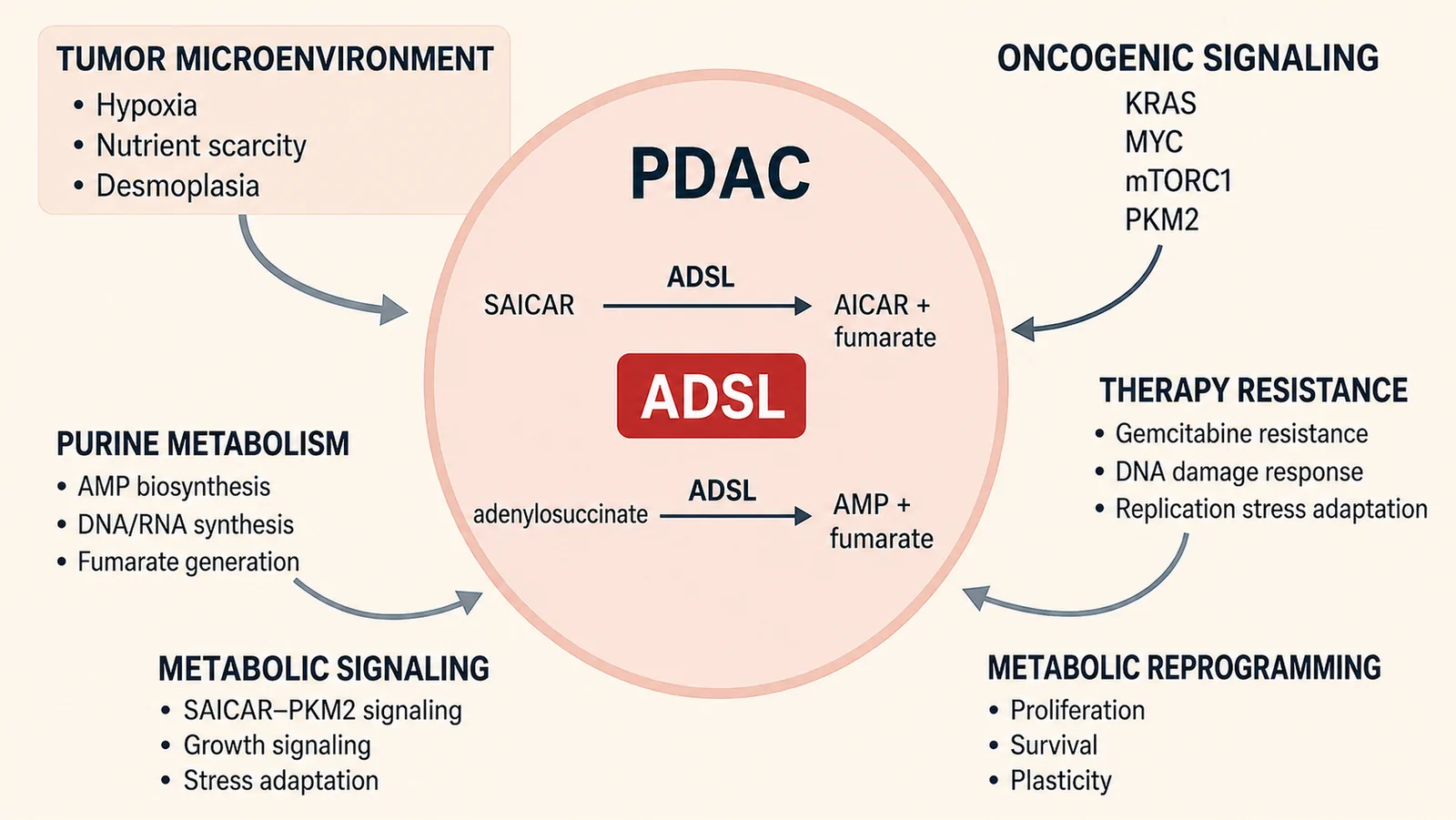
ADSL links oncogenic signaling to metabolic rewiring and chemoresistance in PDAC. This schematic illustrates how oncogenic and stress-adaptive pathways, including KRAS, MYC, mTORC1, and PKM2 signaling, may converge on ADSL-associated purine metabolism to support PDAC progression. ADSL-related metabolic activity contributes to nucleotide-pool maintenance, metabolic plasticity, redox adaptation, and DNA damage tolerance, thereby promoting tumor-cell survival under nutrient limitation and chemotherapeutic stress. ADSL: Adenylosuccinate lyase; PDAC: pancreatic ductal adenocarcinoma; KRAS: KRAS proto-oncogene, GTPase; MYC: MYC proto-oncogene, bHLH transcription factor; mTORC1: mammalian target of rapamycin complex 1; PKM2: pyruvate kinase M2; SAICAR: succinylaminoimidazole carboxamide ribotide; AICAR: aminoimidazole carboxamide ribotide; AMP: adenosine monophosphate.

ADSL upregulation and oncogenic activity have been reported across several malignancies, including prostate, liver, and colorectal cancers^[14-16]^. Altered *de novo* purine biosynthetic flux may influence the balance of pathway intermediates, including SAICAR. SAICAR has been reported to stimulate PKM2 under nutrient-limited conditions^[17]^. However, because SAICAR is a substrate rather than a direct product of ADSL^[8,18,19]^, SAICAR accumulation should not be interpreted as a simple consequence of increased ADSL activity alone, but rather as a context-dependent outcome of altered upstream pathway flux and the balance between SAICAR production and ADSL-mediated consumption. As summarized in Figure 1, SAICAR–PKM2 signaling may link nutrient stress responses to oncogenic transcriptional programs relevant to PDAC metabolism^[20]^. Together with broader evidence that altered metabolic programs contribute to therapeutic resistance in PDAC^[21]^, these findings underscore the importance of defining how ADSL-associated purine metabolism shapes PDAC biology and whether it can be exploited as a therapeutically actionable metabolic vulnerability.

This review summarizes recent advances in our understanding of ADSL in PDAC, with particular emphasis on its metabolic and signaling functions, clinical relevance, and therapeutic potential. We discuss evidence supporting ADSL as a central metabolic node that connects nucleotide biosynthesis with oncogenic signaling, redox adaptation, and tumor microenvironmental remodeling. We also evaluate emerging therapeutic strategies that target ADSL or its downstream consequences to overcome the well-recognized treatment resistance of PDAC and improve clinical outcomes.

## CANONICAL ROLES OF ADSL IN PURINE METABOLISM

ADSL catalyzes two essential reactions in *de novo* purine synthesis^[18,19]^. Specifically, it cleaves SAICAR to AICAR and fumarate and converts adenylosuccinate to AMP and fumarate. Through these reactions, ADSL not only supports the generation of purine nucleotides but also links nucleotide metabolism to the TCA cycle via fumarate production, thereby integrating purine biosynthesis with broader cellular metabolic networks^[8,16,22,23]^.

In non-transformed cells, ADSL expression and enzymatic activity are tightly regulated at both the transcriptional and post-translational levels to maintain purine nucleotide homeostasis and thereby support DNA replication, RNA transcription, and cellular energy balance^[11]^. Appropriate ADSL regulation is also required to maintain genomic stability by ensuring adequate nucleotide availability for DNA replication and repair while preventing the accumulation of potentially toxic intermediates, including SAICAR and adenylosuccinate^[24]^. Consistent with its fundamental physiological importance, inherited ADSL deficiency causes a rare metabolic disorder characterized by neurodevelopmental abnormalities, underscoring the indispensable role of this enzyme in normal cellular function^[25-28]^.

**Figure 2:**
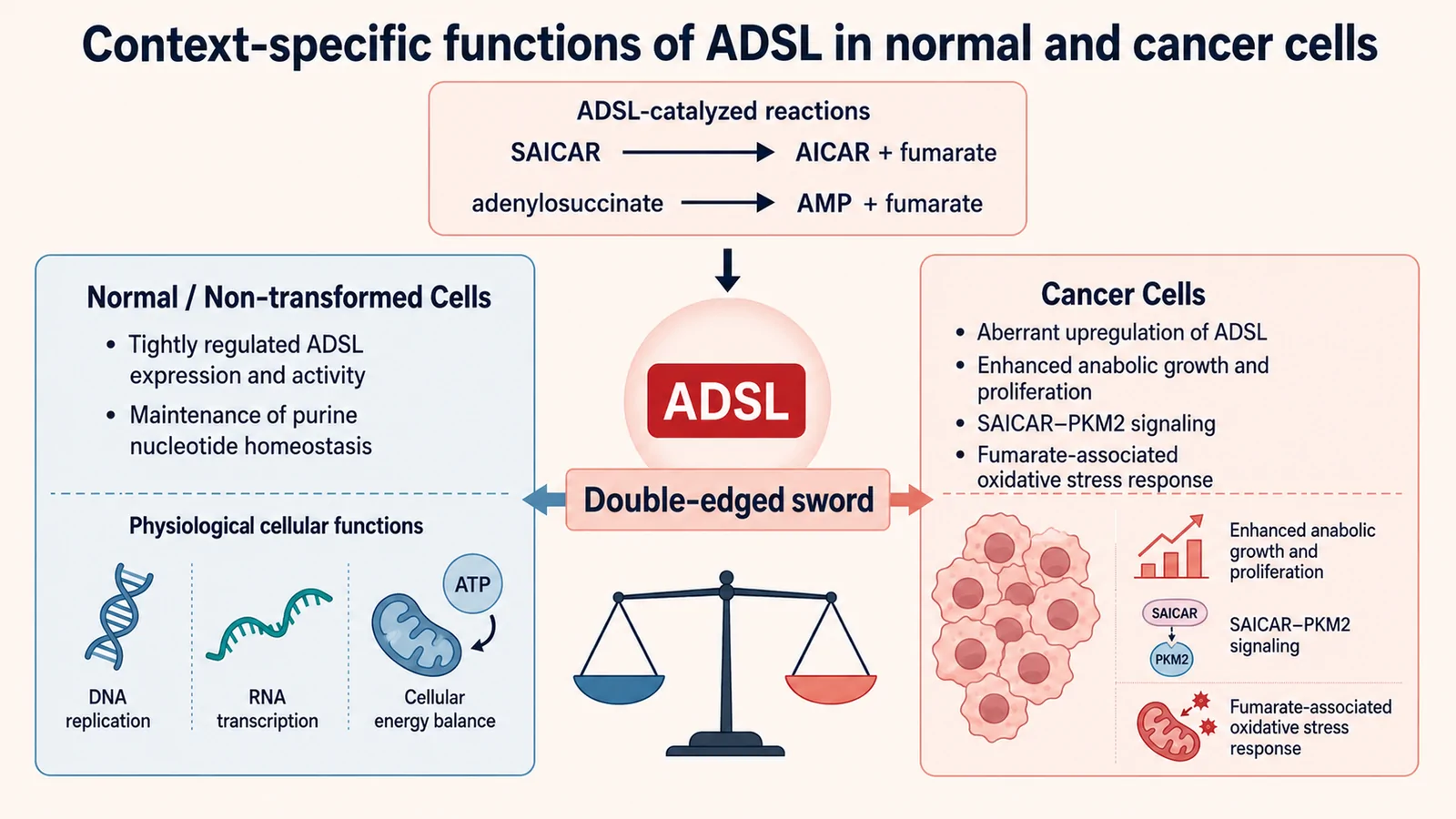
Context-specific functions of ADSL in normal and cancer cells. ADSL maintains purine nucleotide homeostasis in normal cells and thereby supports DNA replication, RNA transcription, and cellular energy balance. In cancer, however, increased ADSL activity may promote anabolic growth, metabolic adaptation, and tumor progression by altering the handling of intermediates such as SAICAR and fumarate. This context dependency highlights the dual role of ADSL as both a physiological metabolic enzyme and a potential driver of malignant phenotypes. ADSL: Adenylosuccinate lyase; SAICAR: succinylaminoimidazole carboxamide ribotide; AICAR: aminoimidazole carboxamide ribotide; AMP: adenosine monophosphate; ATP: adenosine triphosphate; PKM2: pyruvate kinase M2.

However, ADSL also exhibits a “double-edged sword” nature, particularly in cancer biology. Although ADSL is required to maintain physiological purine nucleotide homeostasis, aberrant activation of ADSL-associated *de novo* purine biosynthesis may support malignant progression by sustaining the increased nucleotide demand of rapidly proliferating tumor cells^[11,12,15,16]^. Altered purine biosynthetic flux may also affect the abundance of regulatory pathway intermediates and metabolic by-products. For example, SAICAR has been reported to stimulate PKM2 under nutrient-limited conditions^[17]^. However, because SAICAR is consumed by ADSL during its conversion to AICAR and fumarate, SAICAR accumulation should be regarded as a flux-dependent event rather than a direct consequence of increased ADSL activity. Conversely, fumarate is a catalytic by-product of ADSL-mediated reactions and may contribute to epigenetic remodeling, oxidative stress responses, and immunometabolic regulation depending on cellular context^[13,22,29]^. This duality highlights the need for tumor-selective and context-aware therapeutic strategies when considering ADSL as a target [Figure 2].

## ADSL EXPRESSION AND CANDIDATE CLINICAL RELEVANCE IN PDAC

The clinical relevance of ADSL-associated metabolism in PDAC remains an emerging area. Current PDAC-specific evidence most directly supports a functional role for the NRF2–ADSL axis in gemcitabine responsiveness and ferroptosis-associated adaptation^[9]^. In contrast, robust PDAC-specific evidence demonstrating increased ADSL protein expression by immunohistochemistry or proteomics, or linking ADSL protein abundance to tumor-node-metastasis (TNM) stage, histologic differentiation, metastasis, overall survival (OS), disease-free survival (DFS), or independent prognostic value, remains limited. Therefore, ADSL should currently be regarded as a candidate metabolic marker and mechanistic mediator rather than a clinically validated standalone prognostic biomarker in PDAC.

Mechanistically, the potential oncogenic relevance of ADSL is supported by non-PDAC cancer studies showing that ADSL-associated *de novo* purine biosynthesis can contribute to tumor growth and metabolic adaptation^[15,16]^. More broadly, nucleotide metabolism is a pan-cancer metabolic dependency^[11,12]^, and functional genomic studies in pancreatic cancer have shown that metabolic dependencies can shape pancreatic tumor fitness^[29]^. However, these studies do not establish PDAC-specific clinicopathologic or survival associations for ADSL. Accordingly, future work should validate ADSL expression and activity in clinically annotated PDAC cohorts using transcriptomic, proteomic, immunohistochemical, and metabolomic approaches and should correlate these measurements with treatment response, survival outcomes, and established clinicopathologic variables. Until such validation is available, ADSL expression should be interpreted as a hypothesis-generating biomarker candidate rather than a validated prognostic or predictive marker.

## ADSL-MEDIATED METABOLIC REWIRING IN PDAC

ADSL-associated purine metabolism may contribute to PDAC progression by coupling nucleotide biosynthesis to proliferative and metabolic programs. By sustaining intracellular pools of AMP and other purine nucleotides, ADSL supports DNA replication, RNA transcription, and cellular energy homeostasis, all of which are essential for meeting the high proliferative demands of malignant cells^[22,30-32]^. Beyond this canonical role, ADSL-associated purine metabolism may exert non-canonical effects through flux-dependent pathway intermediates and catalytic by-products, including SAICAR–PKM2 signaling and fumarate-associated pathways, which may engage diverse oncogenic and metabolic networks to reprogram tumor-cell behavior^[33,34]^.

SAICAR is a *de novo* purine biosynthetic intermediate and a substrate of ADSL, which converts SAICAR to AICAR and fumarate. Therefore, increased ADSL expression or activity should not be equated with SAICAR accumulation. Rather, SAICAR accumulation may occur when upstream *de novo* purine biosynthetic flux exceeds ADSL-mediated consumption or when pathway balance is altered under nutrient-limited or stress-adaptive conditions. Under such conditions, SAICAR has been reported to stimulate PKM2, thereby linking *de novo* purine-pathway intermediates to glycolytic regulation and stress adaptation^[17]^. This finding supports a nutrient-stress-dependent SAICAR–PKM2 signaling mechanism. In PDAC, this mechanism may conceptually intersect with metabolic reprogramming and broader oncogenic metabolic plasticity; however, direct evidence that SAICAR–PKM2 signaling drives PDAC-specific cell-cycle progression, biosynthetic output, or chemoresistance remains limited and warrants further validation^[6,35]^.

**Figure 3:**
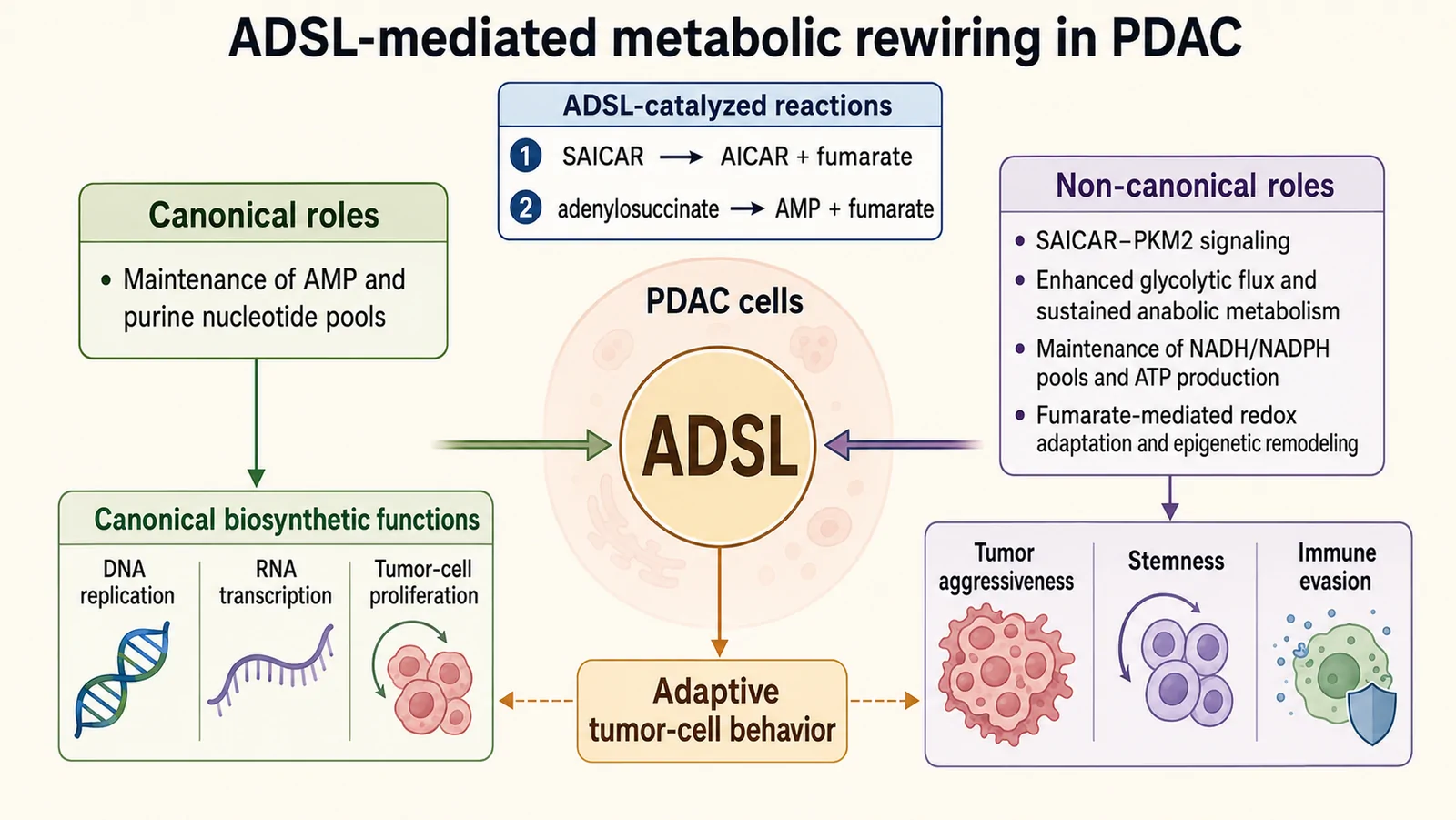
ADSL-mediated metabolic rewiring in PDAC. This figure highlights the non-canonical metabolic outputs of ADSL activity in PDAC. SAICAR–PKM2 signaling may reinforce glycolytic and transcriptional programs, whereas compartment-specific fumarate signaling may contribute to redox adaptation, epigenetic regulation, and innate immune modulation. These pathways position ADSL as a metabolic hub linking purine metabolism with adaptive tumor-cell behavior. ADSL: Adenylosuccinate lyase; PDAC: pancreatic ductal adenocarcinoma; SAICAR: succinylaminoimidazole carboxamide ribotide; PKM2: pyruvate kinase M2; AICAR: aminoimidazole carboxamide ribotide; AMP: adenosine monophosphate; NADH: reduced nicotinamide adenine dinucleotide; NADPH: reduced nicotinamide adenine dinucleotide phosphate; ATP: adenosine triphosphate.

In addition to flux-dependent SAICAR–PKM2 signaling, ADSL-derived fumarate may provide a cytosolic source of fumarate that links *de novo* purine biosynthesis to compartment-specific epigenetic and immunometabolic regulation^[12,13,36]^. Importantly, the biological consequences of fumarate are likely to depend on its subcellular origin and compartmental distribution. Mitochondrial fumarate functions canonically as a TCA-cycle intermediate that supports mitochondrial bioenergetics, oxidative phosphorylation, and redox balance^[10]^. In contrast, ADSL-derived fumarate is generated primarily in the cytosol during *de novo* purine biosynthesis and may influence non-canonical signaling pathways before or after redistribution across subcellular compartments^[12,13]^. Cytosolic fumarate accumulation can promote electrophilic stress and protein modification, whereas nuclear-accessible fumarate may affect alpha-ketoglutarate (α-KG)-dependent dioxygenase activity, including enzymes involved in chromatin and DNA methylation control^[36]^. Thus, ADSL-associated fumarate should not be interpreted simply as equivalent to mitochondrial TCA-cycle fumarate. Rather, its functional impact in PDAC may reflect compartment-specific fumarate signaling that links cytosolic purine metabolism with mitochondrial redox adaptation, nuclear epigenetic regulation, and innate immune modulation. Future studies using subcellular metabolomics and isotope-tracing approaches will be needed to define how ADSL-derived fumarate is partitioned among cytosolic, mitochondrial, and nuclear pools in PDAC cells. Together, these observations position ADSL as a multifaceted metabolic hub that coordinates biosynthetic demand with tumor-adaptive responses [Figure 3].

## GEMCITABINE METABOLISM AND NUCLEOTIDE-POOL ADAPTATION

Gemcitabine is a pyrimidine nucleoside analogue whose cytotoxic activity depends on intracellular transport, metabolic activation, ribonucleotide reductase inhibition, and incorporation into DNA synthesis. After cellular uptake, gemcitabine is phosphorylated by deoxycytidine kinase (dCK) to gemcitabine monophosphate, followed by further conversion to gemcitabine diphosphate and gemcitabine triphosphate. Gemcitabine diphosphate inhibits ribonucleotide reductase, thereby perturbing deoxyribonucleotide pools, whereas gemcitabine triphosphate competes with endogenous deoxycytidine triphosphate for incorporation into DNA and promotes replication arrest and DNA damage. Accordingly, canonical mechanisms of gemcitabine resistance are most directly linked to drug transport, dCK-dependent activation, drug inactivation, ribonucleotide reductase activity, deoxyribonucleotide-pool regulation, DNA damage response (DDR) capacity, and cell-cycle adaptation rather than to purine metabolism alone^[37,38]^.

Within this pharmacologic framework, purine metabolism may influence gemcitabine responsiveness indirectly by shaping nucleotide-pool homeostasis and the capacity of tumor cells to tolerate replication stress. Although gemcitabine primarily targets pyrimidine nucleotide metabolism, previous studies have shown that gemcitabine exposure also induces broader changes in ribonucleotide and deoxyribonucleotide pools. In tumor cell-line models, gemcitabine increased adenosine triphosphate (ATP), guanosine triphosphate (GTP), and other ribonucleotide pools, and similar increases in ATP and GTP pools were also reported in solid tumor models^[39-41]^. In this context, enhanced purine availability may support energy balance, RNA synthesis, DNA-repair-associated nucleotide demand, and adaptive survival during gemcitabine-induced replication stress. Conversely, disruption of *de novo* purine synthesis may limit the ability of tumor cells to maintain ATP/GTP pools and nucleotide balance under chemotherapeutic pressure.

ADSL may therefore modulate gemcitabine responsiveness by affecting the metabolic resilience of purine nucleotide homeostasis rather than by acting as a direct determinant of gemcitabine metabolism. Because ADSL participates in *de novo* purine biosynthesis and contributes to AMP and fumarate generation, altered ADSL activity may influence the ability of PDAC cells to restore adenine nucleotide pools and buffer gemcitabine-induced replication stress. In addition, nucleotide imbalance may affect dCK-dependent gemcitabine activation, as dCK activity and nucleoside analogue phosphorylation are sensitive to intracellular nucleotide context^[42,43]^. Thus, ADSL-associated purine remodeling could intersect with gemcitabine pharmacology through nucleotide-pool balance, dCK-dependent activation, and DNA damage tolerance. However, direct evidence linking ADSL activity to dCK function, gemcitabine triphosphate accumulation, and clinical gemcitabine resistance in PDAC remains limited. Future studies should directly measure ATP/GTP pools, deoxyribonucleotide pools, dCK activity, gemcitabine triphosphate accumulation, SAICAR/AICAR ratios, replication stress, and DNA damage markers following ADSL perturbation and gemcitabine exposure in clinically relevant PDAC models.

The translational relevance of targeting nucleotide metabolism in combination with gemcitabine is also supported by prior studies of gemcitabine with pemetrexed. Pemetrexed is a multitargeted antifolate that inhibits folate-dependent nucleotide biosynthesis, including pathways involved in purine synthesis, and has been evaluated in preclinical and clinical combination strategies with gemcitabine^[44,45]^. These studies provide pathway-level precedent that perturbing nucleotide biosynthesis can interact with gemcitabine efficacy. Nevertheless, pemetrexed is not ADSL-selective, and these findings should be interpreted as indirect support for nucleotide-metabolism-based combination therapy rather than direct evidence for ADSL-targeted treatment in PDAC.

## ADSL IN GEMCITABINE RESPONSIVENESS AND CHEMORESISTANCE

Building on this nucleotide-pool framework, ADSL-associated purine metabolism may modulate, rather than directly determine, gemcitabine responsiveness by influencing the ability of PDAC cells to maintain nucleotide availability, tolerate replication stress, and adapt to chemotherapy-induced metabolic pressure. In PDAC, gemcitabine resistance is closely associated with metabolic adaptation, DDR activation, and replication-stress tolerance^[46,47]^. Direct preclinical evidence indicates that the NRF2–ADSL axis regulates gemcitabine responsiveness in PDAC cells and may be linked to ferroptosis-associated mechanisms^[9]^. Evidence from non-PDAC cancer models further supports the broader concept that *de novo* purine biosynthesis and ADSL activity can sustain nucleotide availability, mitochondrial function, and stress tolerance under metabolic or genotoxic pressure^[15]^. Together, these findings support a context-dependent model in which dysregulation of ADSL-associated metabolism may modulate gemcitabine responsiveness in PDAC, while also highlighting the need to distinguish PDAC-specific evidence from broader cancer-metabolism mechanisms.

In addition to its role in nucleotide metabolism and replication-stress regulation, the NRF2–ADSL axis may influence gemcitabine responsiveness through ferroptosis-associated mechanisms^[9]^. NRF2 is a central transcriptional regulator of antioxidant defense and redox homeostasis, and its activation enables tumor cells to adapt to chemotherapy-induced oxidative and lipid-peroxidative stress^[48,49]^. In gemcitabine-resistant PDAC cells, dysregulation of the NRF2–ADSL axis has been associated with altered redox homeostasis and ferroptotic vulnerability^[9]^. Alteration of this axis may perturb redox homeostasis, lipid peroxidation, and ferroptotic susceptibility during gemcitabine exposure. These findings suggest that ADSL may modulate, rather than uniformly promote, gemcitabine resistance by linking nucleotide metabolism with redox and ferroptosis-associated signaling. However, further validation in patient-derived models and clinically annotated cohorts is required before the NRF2–ADSL axis can be used as a predictive biomarker or therapeutic stratification tool.

**Figure 4:**
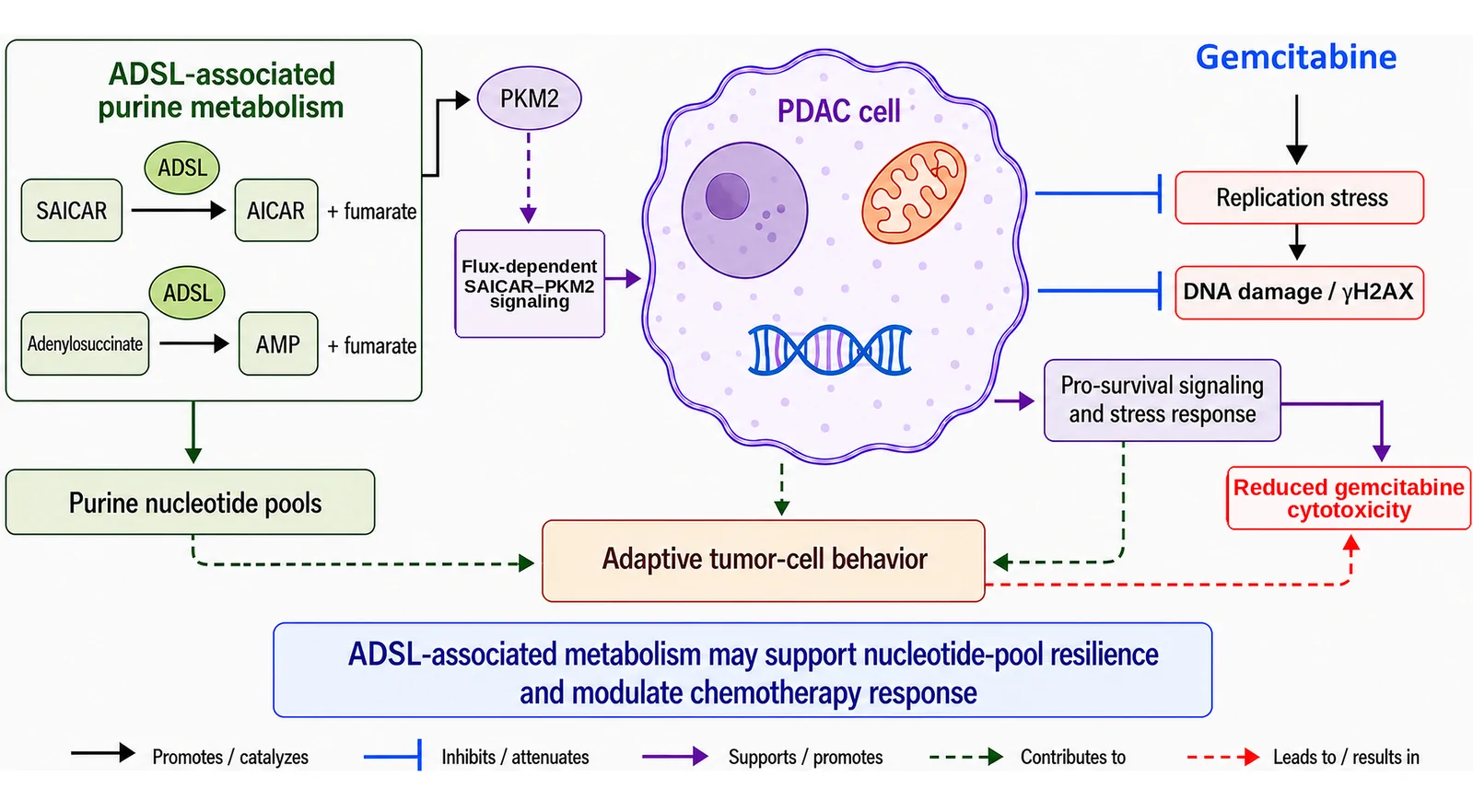
Proposed role of ADSL-associated metabolism in PDAC chemoresistance. ADSL-associated purine metabolism may support *de novo* purine biosynthesis and help maintain nucleotide availability in PDAC cells exposed to chemotherapeutic stress. Through purine nucleotide-pool maintenance, flux-dependent SAICAR–PKM2 signaling, and downstream effects on bioenergetic and biosynthetic programs, ADSL-associated metabolism may facilitate cellular adaptation to antimetabolite treatment. In the setting of gemcitabine exposure, ADSL-associated metabolism is proposed to attenuate replication stress and limit DNA damage, as reflected by reduced gH2AX accumulation, thereby sustaining pro-survival signaling and reducing gemcitabine cytotoxicity. ADSL: Adenylosuccinate lyase; PDAC: pancreatic ductal adenocarcinoma; SAICAR: succinylaminoimidazole carboxamide ribotide; PKM2: pyruvate kinase M2; gH2AX: gamma-H2AX; AICAR: aminoimidazole carboxamide ribotide; AMP: adenosine monophosphate.

Preclinical evidence supports the concept that modulation of ADSL-associated metabolism may alter gemcitabine responsiveness in PDAC models^[9]^. Tumors with distinct ADSL expression patterns or *de novo* purine-metabolic dependencies may therefore represent metabolically defined subgroups that require different ADSL-associated therapeutic strategies. In addition, targeting ADSL-associated nucleotide metabolism may be rationally combined with DDR inhibitors, including ataxia telangiectasia and Rad3-related (ATR) or poly(ADP-ribose) polymerase (PARP) inhibitors, to intensify replication stress and overwhelm residual DNA-repair capacity^[47]^. These findings support a rationale for combination strategies that pair ADSL-directed metabolic intervention with cytotoxic chemotherapy or targeted DDR blockade [Figure 4].

Despite these opportunities, therapeutic targeting of ADSL *in vivo* presents important challenges. Because ADSL is required for nucleotide homeostasis in normal proliferative tissues such as bone marrow and intestinal epithelium, systemic inhibition may produce dose-limiting toxicities. Accordingly, tumor-selective strategies, including salvage-pathway co-targeting, synthetic-lethal approaches in defined genetic contexts such as methylthioadenosine phosphorylase (MTAP) deficiency, and nanoparticle-based drug delivery, are being explored to improve selectivity and reduce collateral injury^[50]^. In addition, the marked metabolic plasticity of PDAC may permit compensatory activation of purine salvage pathways or parallel pyrimidine programs, underscoring the need for comprehensive metabolic profiling to anticipate resistance mechanisms^[35,51,52]^. Overall, current evidence supports a context-dependent model in which dysregulated ADSL-associated metabolism may modulate chemoresistance by altering nucleotide availability, genotoxic-stress adaptation, and redox homeostasis. The therapeutic consequences of targeting ADSL are therefore likely to depend on the metabolic state of individual PDAC tumors and require further experimental validation^[53]^.

## *DE NOVO* PURINE SYNTHESIS *VS.* SALVAGE PATHWAY COMPENSATION IN PDAC

A critical issue in evaluating ADSL as a therapeutic vulnerability is whether PDAC cells primarily depend on *de novo* purine synthesis or can compensate through purine salvage pathways under therapeutic stress. *De novo* purine biosynthesis supports nucleotide production required for DNA replication, RNA synthesis, and repair of chemotherapy-induced DNA damage^[11,19]^. In tumors that are functionally dependent on ADSL-driven *de novo* purine synthesis, ADSL inhibition may increase nucleotide stress and enhance sensitivity to antimetabolite-based therapy. In addition, oncogenic signaling can regulate *de novo* purine synthesis flux through enzymes such as PFAS, further supporting the concept that purine biosynthesis is dynamically coupled to tumor growth and adaptive stress responses^[54]^. However, PDAC is characterized by pronounced metabolic plasticity, and tumor cells may engage compensatory salvage mechanisms to maintain purine nucleotide pools when *de novo* synthesis is impaired^[6,55]^. Enzymes such as hypoxanthine phosphoribosyltransferase 1 (HPRT1) and adenine phosphoribosyltransferase (APRT) can recycle purine bases and may partially rescue nucleotide availability after ADSL suppression^[55]^. Therefore, the therapeutic efficacy of ADSL inhibition may depend not only on ADSL expression or *de novo* purine flux, but also on the activity of purine salvage pathways and the availability of extracellular nucleosides or bases within the nutrient-restricted PDAC microenvironment. Future studies should incorporate metabolic-flux analysis, SAICAR/AICAR profiling, AMP/IMP/GMP quantification, and assessment of HPRT1/APRT activity to define whether individual PDAC tumors are *de novo* synthesis-dependent, salvage-dependent, or metabolically flexible. Such stratification will be essential for determining whether ADSL inhibition should be used alone or combined with salvage-pathway co-targeting.

## STRATEGIES FOR THERAPEUTICALLY TARGETING ADSL IN PDAC

Given its central role in tumor cell metabolism, DNA synthesis, and survival under stress, ADSL has emerged as a candidate therapeutic target in metabolically aggressive malignancies^[15,16,56]^. In PDAC, the strong dependence of tumor cells on adaptive metabolic programs, nucleotide stress tolerance, and DDR pathways supports the rationale for investigating ADSL-associated purine metabolism as a potential metabolic vulnerability, although further validation in clinically relevant models is required^[29,35,47]^. Inhibiting ADSL disrupts *de novo* purine biosynthesis, thereby compromising the tumor’s ability to maintain nucleotide pools, repair DNA, and tolerate genotoxic stress^[57,58]^. Because ADSL-associated metabolism may intersect with broader pro-tumorigenic networks, including mTORC1–MYC signaling, KRAS proto-oncogene, GTPase (KRAS)-driven metabolic flux, and redox homeostasis, it may represent a convergence point for therapeutic intervention^[16,48,49,59]^. Genetic ADSL depletion has shown antitumor effects in selected non-PDAC models; however, clinically validated ADSL-selective pharmacologic inhibitors remain unavailable. Potential strategies to improve selectivity include tumor-targeted delivery, salvage-pathway co-targeting, and synthetic-lethal approaches in genetically defined settings such as MTAP-deficient tumors; however, these approaches remain conceptual or preclinical in the context of ADSL-directed therapy in PDAC^[50,55,60]^. To clarify the translational landscape of ADSL-directed therapy, current ADSL-targeting and related purine-metabolic intervention strategies are summarized in Table 1. Because clinically established ADSL-selective inhibitors remain unavailable, the table includes both direct and indirect strategies relevant to ADSL-associated metabolic vulnerabilities.

**Table 1:** Direct, indirect, and conceptual strategies targeting ADSL-associated purine-metabolic vulnerabilities in PDAC

**Approach**	**Strategy/agent**	**Target or mechanism**	**Evidence status**	**PDAC relevance**	**Status/limitation**	**Refs.**
Direct ADSL targeting	Genetic ADSL depletion or future ADSL-selective inhibitors	ADSL expression or catalytic activity	Preclinical genetic evidence, mainly from non-PDAC models	Relevant to functionally validated ADSL-dependent tumors with high *de novo* purine flux	No clinically approved or validated clinical-stage ADSL-selective inhibitor; efficacy in ADSL-low, gemcitabine-resistant tumors remains uncertain	[15,16]
Indirect pathway-level targeting	L-alanosine	ADSS and *de novo* AMP biosynthesis; not ADSL-selective	Historical clinical development, including a phase II study in MTAP-deficient cancers	Provides pathway-level precedent for targeting adenine nucleotide synthesis; not PDAC-specific	Limited historical clinical activity and toxicity concerns; no biomarker-defined evidence in PDAC	[50]
Indirect salvage-pathway co-targeting	Genetic or pharmacologic targeting of HPRT1 and/or APRT	Purine salvage pathway	Pathway-supported preclinical evidence; ADSL combination remains conceptual	May limit compensatory purine salvage after suppression of *de novo* synthesis	No direct evidence for ADSL–HPRT1/APRT co-targeting in PDAC; metabolic-flux validation is required	[55,62]
Conceptual combination strategy	ATR/PARP inhibitors with state-matched ADSL modulation	Replication-stress and DNA-damage-response pathways	Conceptual for ADSL combination; independent PDAC preclinical evidence supports gemcitabine plus ATR inhibition	May enhance replication stress in purine-dependent PDAC tumors	No direct evidence for ADSL–ATR/PARP combinations in PDAC; efficacy and toxicity remain undefined	[47]
Conceptual enabling strategy	Nanoparticle- or receptor-targeted delivery of ADSL-directed agents	Tumor-selective delivery rather than ADSL biology itself	No ADSL-specific study; general PDAC nanoparticle and siRNA delivery proof-of-concept	May improve tumor delivery and reduce systemic toxicity of ADSL-directed interventions	Preclinical only; no established ADSL-targeted delivery platform or clinical evidence in PDAC	[63,64]

ADSL: Adenylosuccinate lyase; PDAC: pancreatic ductal adenocarcinoma; ADSS: adenylosuccinate synthetase; AMP: adenosine monophosphate; MTAP: methylthioadenosine phosphorylase; HPRT1: hypoxanthine phosphoribosyltransferase 1; APRT: adenine phosphoribosyltransferase; ATR: ataxia telangiectasia and Rad3-related; PARP: poly(ADP-ribose) polymerase; siRNA: small interfering RNA.

A particularly attractive direction is developing rational combination regimens that exploit vulnerabilities created by ADSL inhibition. Co-targeting the purine salvage pathway, for example through HPRT1 or APRT, may suppress compensatory rescue mechanisms and thereby enhance cytotoxicity^[55]^. In functionally validated ADSL-dependent tumors, ADSL inhibition may theoretically enhance sensitivity to DDR inhibitors such as ATR and PARP inhibitors by amplifying replication stress and reducing repair capacity; however, direct evidence for ADSL–DDR inhibitor combinations in PDAC remains limited^[47]^. Because ADSL-associated fumarate may also contribute to compartment-specific redox and stress-adaptive signaling, additional synergy with redox-directed therapies remains a testable hypothesis. Such multidimensional strategies are especially relevant in PDAC, where metabolic plasticity, stromal restriction, and adaptive resistance frequently limit the durability of single-agent approaches^[6,35]^.

In this setting, metabolomic profiling may help refine patient stratification and therapeutic monitoring by identifying tumors with functional ADSL dependency, high *de novo* purine-synthesis activity, or limited salvage-pathway compensation^[6,11,12,35]^. Tumor-selective delivery systems could further expand the therapeutic window by reducing off-target toxicity. Molecular and metabolic stratification based on functional ADSL dependency, *de novo* purine-synthesis activity, salvage-pathway capacity, or MTAP loss may help identify patient subsets that are more likely to benefit from ADSL-associated metabolic interventions^[55,61]^. In parallel, companion diagnostics, including metabolite-based imaging platforms and circulating purine-related biomarkers, may guide treatment selection and permit dynamic assessment of response. Although ADSL dysregulation may be associated with altered gemcitabine responsiveness, ADSL should currently be regarded as a candidate biomarker rather than a clinically validated standalone predictor of treatment response^[9]^. Gemcitabine response in PDAC is multifactorial and may be influenced by nucleotide metabolism, drug uptake and activation, DDR capacity, stromal drug-delivery barriers, and treatment context^[6,11]^. Therefore, ADSL expression or activity may be more informative when integrated with complementary metabolic and pharmacologic markers, such as SAICAR/AICAR ratios, fumarate-associated signatures, purine salvage activity, and established determinants of gemcitabine metabolism. Prospective validation in clinically annotated PDAC cohorts and patient-derived models will be required before ADSL can be implemented for patient stratification.

Beyond tumor-intrinsic metabolic stress, ADSL may also contribute to immune evasion through fumarate-mediated suppression of innate immune signaling. Recent evidence indicates that ADSL-generated fumarate can directly bind to and post-translationally modify stimulator of interferon genes (STING), thereby impairing STING activation and downstream cyclic GMP-AMP synthase–stimulator of interferon genes (cGAS–STING) signaling^[13]^. Because STING activation normally promotes TANK-binding kinase 1 (TBK1)–interferon regulatory factor 3 (IRF3) signaling, type I interferon production, interferon-stimulated gene expression, and antitumor immune surveillance, fumarate-mediated STING inhibition may reduce innate immune activation and facilitate immune escape. This mechanism may be particularly relevant in PDAC, which is widely characterized as an immunologically “cold” tumor type with dense desmoplastic stroma, limited cytotoxic T-cell infiltration, abundant immunosuppressive myeloid and stromal compartments, and poor responsiveness to immune checkpoint blockade^[4,35]^. Therefore, ADSL inhibition may have immunometabolic consequences beyond direct tumor-cell metabolic stress and could potentially enhance STING-dependent inflammatory signaling or improve responsiveness to immune-based combination strategies. However, the direct contribution of the ADSL–fumarate–STING axis in PDAC remains to be fully validated in immunocompetent and patient-derived models.

**Figure 5:**
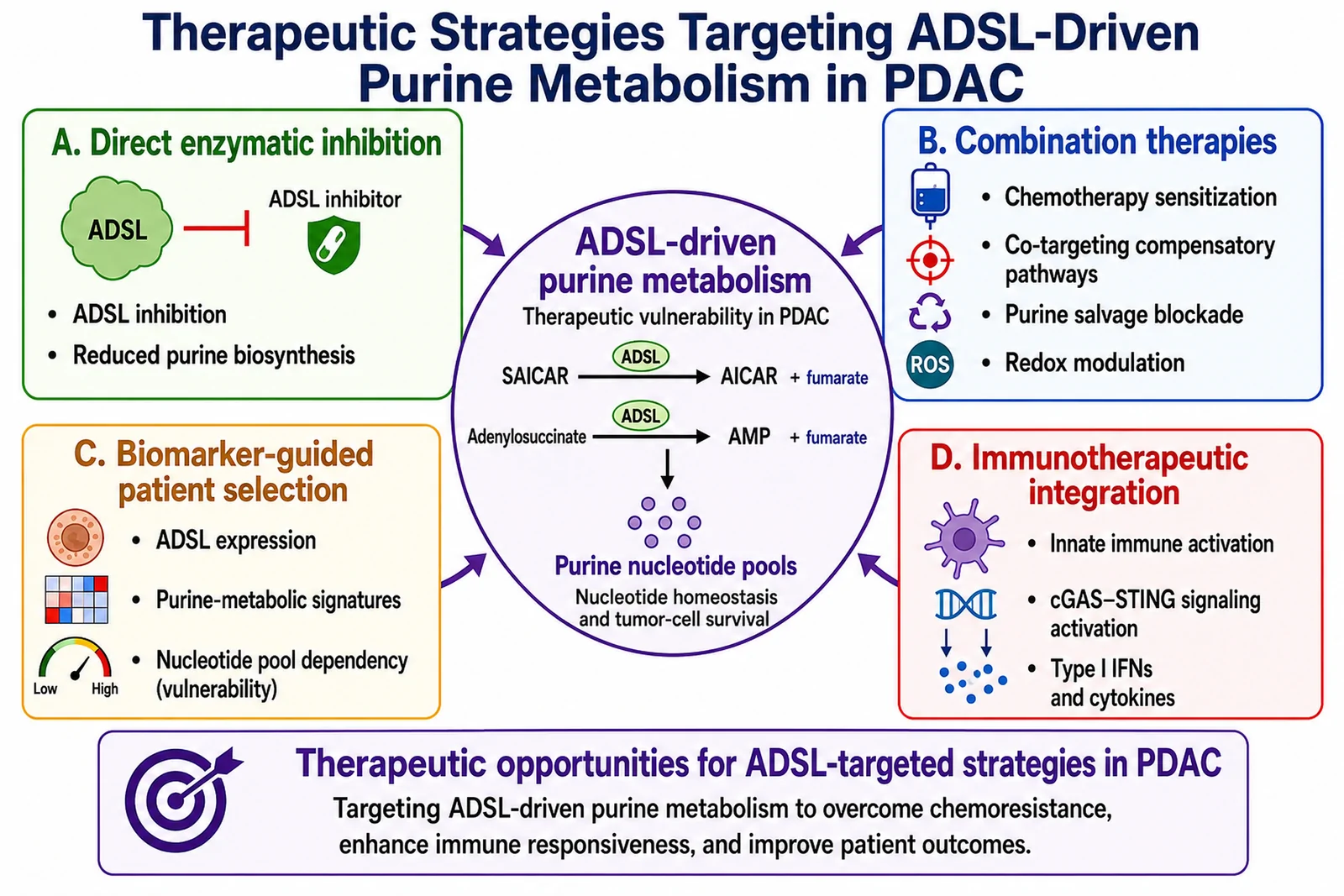
Therapeutic strategies targeting ADSL-driven purine metabolism in PDAC. This schematic summarizes translational strategies for targeting ADSL-associated metabolic vulnerabilities. Four complementary approaches are shown: (A) direct or tumor-selective ADSL inhibition; (B) rational combination therapy targeting purine salvage, DDR pathways, redox adaptation, or chemotherapy resistance; (C) biomarker-guided patient selection using ADSL expression and purine-metabolic features; and (D) immunotherapeutic integration through modulation of innate immune signaling, including the cGAS–STING axis. ADSL: Adenylosuccinate lyase; PDAC: pancreatic ductal adenocarcinoma; DDR: DNA damage response; cGAS–STING: cyclic GMP-AMP synthase–stimulator of interferon genes; SAICAR: succinylaminoimidazole carboxamide ribotide; AICAR: aminoimidazole carboxamide ribotide; AMP: adenosine monophosphate; ROS: reactive oxygen species; IFNs: interferons.

Ultimately, integrating ADSL inhibition into combination regimens that include chemotherapy, DDR inhibitors, redox-directed therapies, or immune checkpoint blockade may provide a strategy to overcome the therapeutic resistance and immune exclusion that characterize PDAC [Figure 5]. Collectively, these advances will be crucial for translating ADSL from a mechanistic concept into a potentially actionable target in precision oncology.

## IMPORTANCE AND LIMITATIONS

In this review, we provide a mechanistically integrated and translationally oriented perspective on the role of ADSL in PDAC. While previous studies have largely examined purine metabolism or therapeutic resistance in isolation, our synthesis positions ADSL as a central metabolic node linking nucleotide biosynthesis with oncogenic signaling, redox homeostasis, and adaptive responses to therapeutic stress.

A key strength of this review lies in framing ADSL as a context-dependent regulator, rather than a purely anabolic enzyme. We focus on three interconnected dimensions: (i) canonical and non-canonical functions of ADSL in metabolic rewiring, including SAICAR–PKM2 signaling and fumarate-mediated epigenetic and redox regulation; (ii) its context-dependent role in modulating chemoresistance through alterations in nucleotide homeostasis, replication-stress adaptation, and redox regulation; and (iii) emerging therapeutic strategies targeting ADSL-associated vulnerabilities, particularly rational combination approaches with DDR inhibitors and metabolic co-targeting. Importantly, we deliberately limit the scope of this review to prioritize mechanistic depth and translational relevance, rather than providing a comprehensive overview of PDAC metabolism. By integrating evidence from biochemical studies, cancer metabolism research, and preclinical models, we propose a cohesive framework linking ADSL activity to clinically relevant phenotypes, including therapeutic resistance and tumor adaptability.

**Figure 6:**
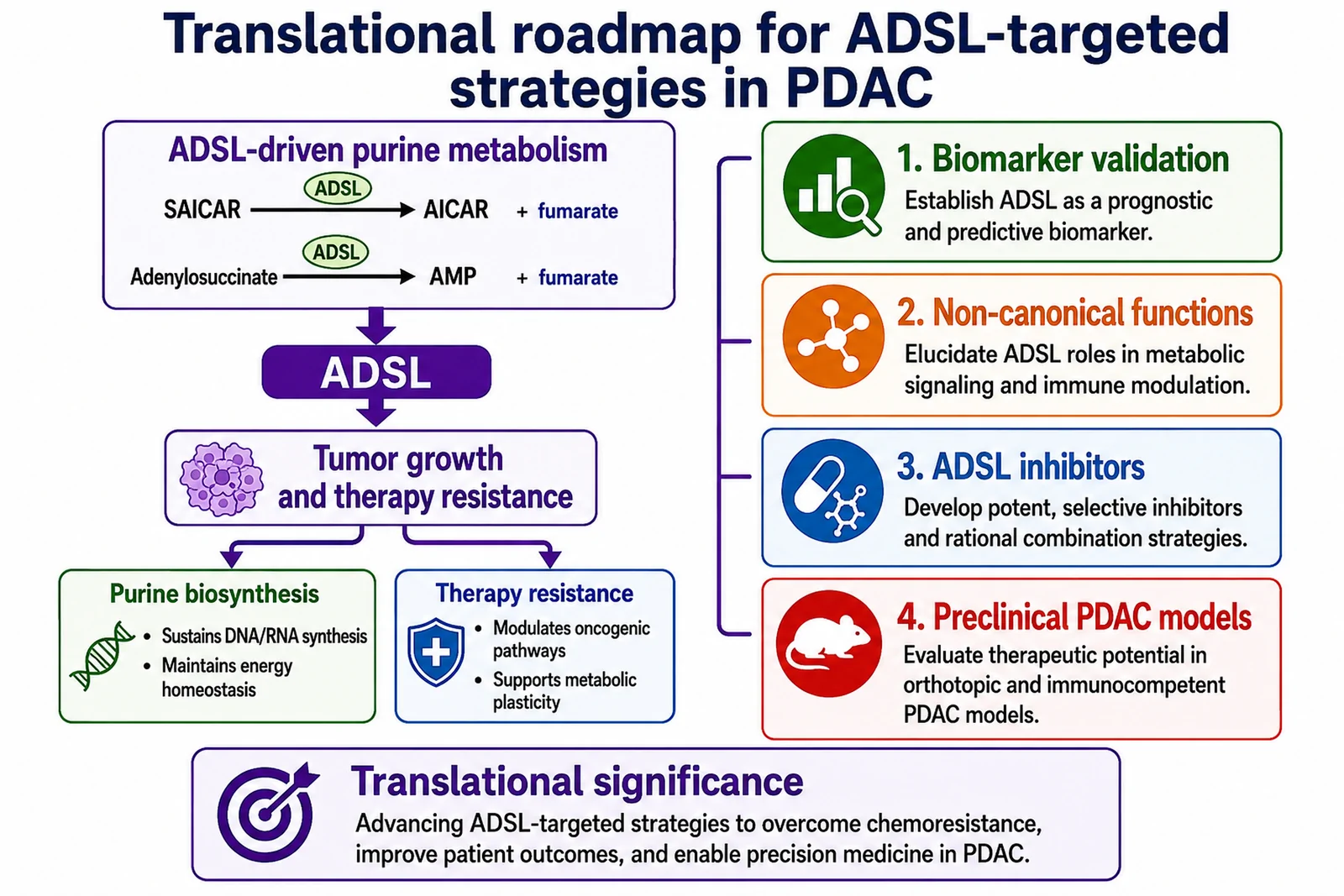
Translational roadmap for advancing ADSL-targeted strategies in PDAC. Future development of ADSL-directed therapy will require integration of mechanistic, biomarker, and preclinical validation studies. Key priorities include the design of potent and tumor-selective ADSL inhibitors, identification of predictive biomarkers such as SAICAR/AICAR ratios or fumarate-associated signatures, and validation in orthotopic and immunecompetent PDAC models. This framework highlights how ADSL may be advanced from a mechanistic metabolic target to a clinically actionable vulnerability in precision oncology. ADSL: Adenylosuccinate lyase; PDAC: pancreatic ductal adenocarcinoma; SAICAR: succinylaminoimidazole carboxamide ribotide; AICAR: aminoimidazole carboxamide ribotide; AMP: adenosine monophosphate.

Despite these advances, several limitations remain. First, the context-dependent role of ADSL - potentially functioning as both a tumor promoter and a metabolic vulnerability - remains insufficiently defined, particularly across molecularly heterogeneous PDAC subtypes. Second, a major translational limitation is that most current evidence supporting ADSL-directed strategies is derived from two-dimensional cell culture systems or simplified *in vitro* models, which cannot fully recapitulate nutrient gradients, stromal restriction, immune-cell exclusion, vascular heterogeneity, or drug-delivery barriers in PDAC. Patient-derived organoids (PDOs) may better preserve patient-specific metabolic dependencies and treatment-response heterogeneity, making them useful for evaluating whether ADSL-high or purine-dependent tumors are more sensitive to ADSL-directed interventions. However, conventional PDO systems often lack intact stromal, vascular, and immune compartments. Patient-derived xenografts (PDXs) may retain tumor architecture and interpatient heterogeneity more faithfully than conventional cell-line models, but they are typically established in immunodeficient hosts and therefore remain limited for assessing ADSL-related immunometabolic effects. In contrast, orthotopic models and genetically engineered mouse models (GEMMs) may better capture tumor–stroma–immune interactions and the dense desmoplastic microenvironment that restricts therapeutic delivery in PDAC. Therefore, future studies should evaluate ADSL inhibition across complementary platforms, including PDOs, PDXs, orthotopic models, and immunocompetent GEMMs, to define therapeutic efficacy, stromal penetration, immune consequences, and toxicity in a clinically relevant context^[6,35]^. Third, the pronounced metabolic plasticity of PDAC may enable compensatory activation of purine salvage or parallel metabolic pathways, potentially limiting the durability of ADSL-targeted therapies and underscoring the necessity for rational combination strategies. Overall, this review advances current understanding by positioning ADSL within a broader metabolic–signaling–therapeutic axis and by proposing a conceptual framework that links metabolic enzyme function to treatment vulnerability. This perspective may facilitate biomarker-guided therapeutic strategies and support the translation of ADSL from a mechanistic concept to a potentially actionable target in precision oncology. These priorities are summarized in a translational roadmap that integrates mechanistic validation, biomarker development, clinically relevant model selection, and therapeutic translation of ADSL-targeted strategies in PDAC [Figure 6].

## CONCLUSIONS AND FUTURE DIRECTIONS

ADSL is increasingly recognized as a central regulator of purine metabolism and an important integrator of oncogenic signaling, redox homeostasis, and therapeutic adaptation in PDAC. Its dual identity as both a determinant of tumor fitness and a context-dependent metabolic vulnerability makes it an attractive candidate for precision oncology. Beyond sustaining nucleotide pools required for proliferation and DNA repair, ADSL also appears to connect metabolism with broader tumor-promoting programs, extending its relevance beyond conventional anabolic function.

Future work should prioritize the development of context-specific strategies targeting ADSL-associated metabolism. These strategies may include tumor-selective ADSL inhibition in functionally validated ADSL-dependent tumors, as well as targeting downstream redox, ferroptosis-associated, or compensatory purine salvage pathways in tumors with distinct ADSL-associated metabolic states. Integrated multi-omic profiling and validation in orthotopic and immunocompetent PDAC models will be essential to define the immunometabolic consequences of ADSL-targeted interventions and evaluate their potential integration with chemotherapy, DDR inhibitors, redox-directed therapies, or immunotherapy. Overall, ADSL-associated metabolism represents a mechanistically informative and potentially actionable vulnerability whose therapeutic relevance likely depends on the tumor-specific metabolic context.

## DECLARATIONS

### AI and AI-assisted tools statement

During the preparation of this manuscript, ChatGPT (OpenAI; GPT-5.5 Thinking; released April 23, 2026) was used to assist with language editing, text polishing, and visual/layout refinement suggestions for Figures 1-6. The AI tool was not used to generate scientific data, perform data analysis, create original experimental results, determine scientific conclusions, or replace authorial control over the figure content. AI assistance for Figures 1-6 was limited to visual/layout refinement suggestions. The authors reviewed, edited, and approved all scientific content, pathway relationships, figure labels, and visual elements. All authors take full responsibility for the accuracy, integrity, originality, and final content of the manuscript.

### Copyright

© The Author(s) 2026.
